# Unrevealed genetic diversity of GII Norovirus in the swine population of North East Italy

**DOI:** 10.1038/s41598-020-66140-4

**Published:** 2020-06-08

**Authors:** L. Cavicchio, L. Tassoni, A. Laconi, G. Cunial, L. Gagliazzo, A. Milani, M. Campalto, G. Di Martino, M. Forzan, I. Monne, M. S. Beato

**Affiliations:** 1 Diagnostic Virology Laboratory, Department of Animal Health, Istituto Zooprofilattico Sperimentale delle Venezie (IZSVe), Viale dell’Università 10, 35020 Legnaro, Padua Italy; 2 EU, OIE/FAO and National Reference Laboratory for Avian Influenza and Newcastle Disease, Istituto Zooprofilattico Sperimentale delle Venezie (IZSVe), Viale dell’Università 10, 35020 Legnaro, Padua Italy; 3 Epidemiology Department, Istituto Zooprofilattico Sperimentale Delle Venezie (IZSVe), Viale dell’Università 10, 35020 Legnaro, Padua Italy; 4 Department of Veterinary Virology, University of Pisa, Viale delle Piagge 2, 56124 Pisa, Italy; 5 Department of Comparative Biomedicine and Food Science, University of Padua, Legnaro, Padua, Italy

**Keywords:** Genetics, Microbiology

## Abstract

Noroviruses (NoVs) are one of the major causative agents of non-bacterial gastroenteritis in humans worldwide. NoVs, belonging to *Caliciviridae*, are classified into ten genogroups (G) and eight P-groups based on major capsid protein (VP1) and of the RNA-dependent-RNA-polymerase (RdRp), respectively. In swine, the main genogroup and P-group identified are GII and GII.P; which can infect humans too. To date, only one case of GIIP.11 have been identified in swine in Italy while the circulation of other P-types is currently unknown. In the present study, 225 swine faecal samples were collected from 74 swine herds in Veneto region through on-farm monitoring. NoV circulation was particularly high in older pigs. The phylogenetic analysis showed the co-circulation of NoVs belonging to two different P-types: GII.P11 and GII.P18, here described for the first time in Italy, presenting an extensive genetic diversity, never described before worldwide. Distinct NoV genetic subgroups and unique amino acid mutations were identified for each P-type for the first time. This study demonstrated the co-circulation of diverse swine NoVs subgroups in Italy, raising questions on the origin of such diversity and suggesting that continuous monitoring of swine NoVs is needed to track the emergence of potentially zoonotic viruses by recombination events.

## Introduction

Food- and water-borne infections, which are mainly manifested with gastroenteritis in humans, represent a significant public health burden. Among food-borne viruses, Noroviruses (NoVs) are a leading pathogen, causing epidemic gastroenteritis cases worldwide^1^. Low infectious dose, environmental resistance, strain diversity, and the shedding from asymptomatic persons, render human NoVs highly contagious pathogens. NoVs are transmitted through ingestion of contaminated food or water, either via the faecal-oral route or via air-borne particles and contact with contaminated surfaces. NoV belongs to the *Caliciviridae* family, which includes small non-enveloped viruses of approximately 35 nm in diameter with single stranded positive RNA genome of 7.4–8.3 kb^2^. Five genera: Vesivirus, Lagovirus, Sapovirus, Nebovirus and Norovirus as well as unassigned viruses are included in this family^3^. Norovirus and Sapovirus genera contain human and animal enteric viruses. The NoV genome possesses 3 Open Reading frames (ORFs) encoding: a polyprotein (ORF1) that by protease processing produces six non-structural proteins, including a RNA-dependent-RNA-polymerase (RdRp), a major capsid protein, VP1 (ORF2), and a minor one, VP2 (ORF3). NoVs are classified into ten genogroups (G) based on the variation of the major capsid protein (VP1), and are further divided into 49 genotypes^3^. Due to the recombination events that occur between ORF1 and ORF2, a dual nomenclature based on VP1 and RdRp was recently proposed^3^. Based on the RdRp sequence, NoVs are classified into eight P (polymerase)-groups and further divided into 60 P-types.

Human NoV infections are caused by GI, GII, and GIV genotypes, with a higher frequency of the GII genotype^4^. Particularly GII.P4 causes up to 85% of human gastroenteritis epidemics worldwide^5^.

Animal enteric caliciviruses genetically related to human NoVs have been detected in pigs and cows^6–9^. In particular, NoVs detected in pigs were classified mainly in three distinct GII P-types (GII.P11, GII.P18, GII.P19)^8–24^. Since the first report of GII NoVs in pigs in the USA, other countries in Europe and Latin America have reported the presence of this genogroup in diseased and healthy pigs^8,14,18,19,25,26^. Detection of GII and GI NoVs in swine fecal samples, retail and imported raw meat samples^7,13,27^, has raised public health concerns about the zoonotic potential of porcine NoVs and the role of swine in the epidemiology of this infection, as a possible source of new viral recombinant strains that can be transmitted directly to humans^7,26,28^. To date, the contribution of swine NoVs to human infections remains unclear and to be elucidated.

Our previous pilot survey on NoVs in swine conducted at slaughterhouses has revealed the presence of GII.P11 in one North East Italian region, Veneto, in 2017^29^. To further elucidate the presence of NoVs in swine in the same region, which is one of the densely populated pig region in Italy, and assess the circulation of other P-groups and/or P-types, a field investigation, with a different sampling strategy was conducted between 2018 and 2019. We tested 225 swine faecal samples collected from 2018 to 2019 to detect swine NoVs and assess their genetic diversity and relatedness. Based on our investigation, we demonstrate that two NoVs P-types co-circulate in pigs in Italy, GII.P11 and GII.P18, with GII.P11 possessing a considerable genetic diversity, and GPII.18 here described for the first time, in Italy. Our study indicates that NoV circulation is higher in fattening farms and in animals>90 days old and that NoV P-type is not associated with herd typology.

## Results

### Distribution and prevalence of NoVs in swine farms

**Figure 1:**
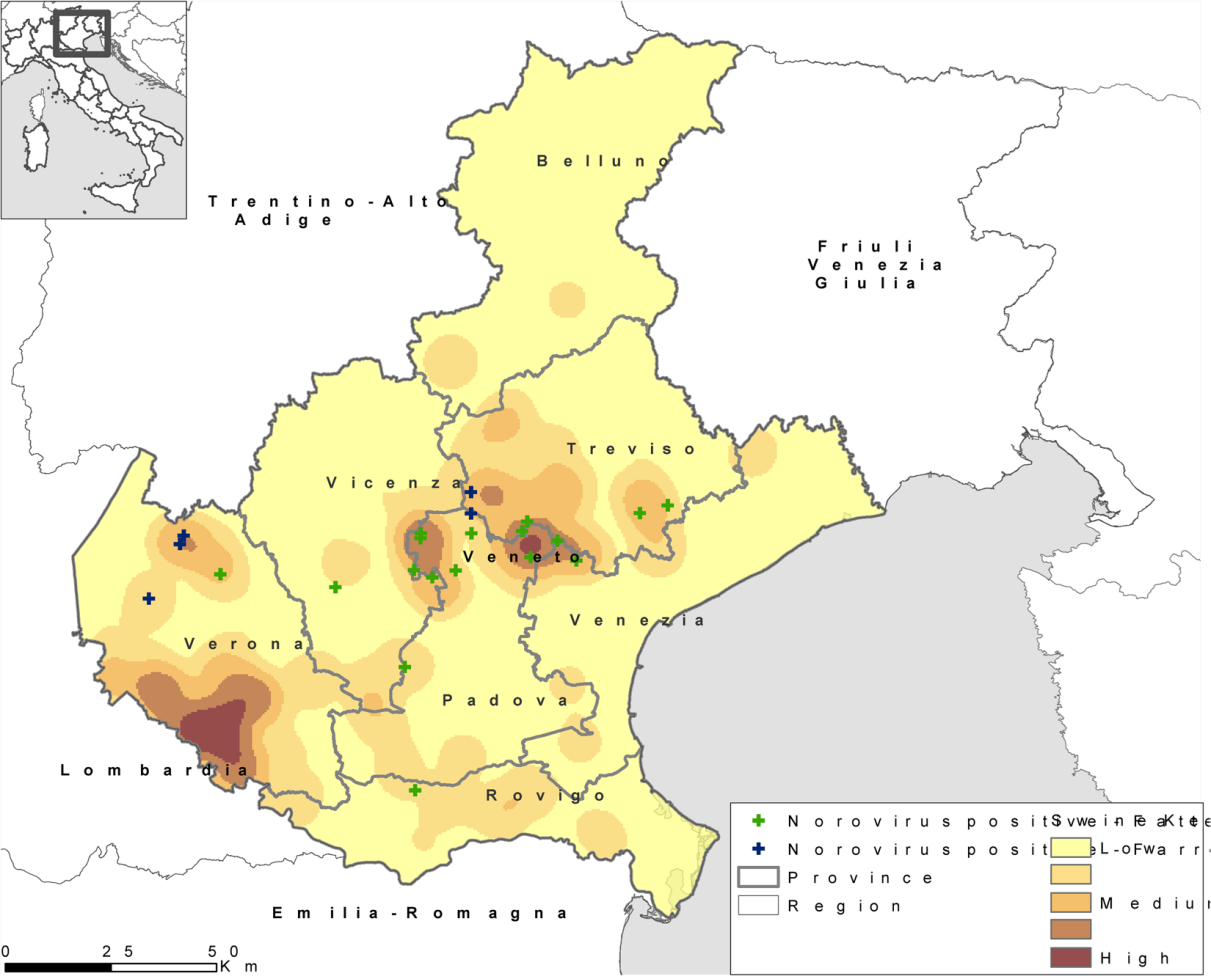
Density map (Kernel Density) of Veneto swine farms and distribution of NoV positive swine farms: 2018–2019. Green crosses identify the NoV positive fattening farms, dark blue crosses the NoV positive farrow to finish farms. The Kernel Density tool calculates the density of features in a neighbourhood around those features. The input data was the industrial swine farms in Veneto region and the density was calculated considering the potential capacity of each farm. The parameter used to calculate the density was a radius of 500 m and the raster cell of the analysis was 10 km^2^.

Between 2018 and 2019, 74 swine herds, of which 43 fattening farms, ten farrow to wean and 21 farrow to finish farms, were sampled, in the Veneto region, collecting a total of 225 swine faeces pool samples (Table 1). Forty-nine out of 225 faeces samples (21.78%) resulted positive for *Caliciviridae*, of which 29 were characterized as NoV (11.4%). The NoV positive faeces were collected in 22 different farms, of which sixteen fattening and six farrow to finish (Table 1). In detail, between January and June 2018 (first-stage sampling), 37 swine farms were sampled and 125 faecal samples collected. The swine herds were located in five out of seven Veneto provinces in densely populated pig areas (located in Verona, Treviso and Padua provinces) for a total of sixteen fattening, ten farrow to wean, and eleven farrow to finish farms (Fig. 1). At this stage, all animal age groups were included. During this first sampling stage, fourteen out of 125 faecal samples (11.2%) resulted NoV positive, distributed in eleven out of 37 farms, of which 7/16 fattening and 4/11 farrow to finish farms. The fourteen NoVs belonged to the GII.P11 (10/14) and GII.P18 P-types (4/14). Thirteen out of fourteen NoV positive faeces samples were identified in fattening animals (90 to 180 days old animals) and one in gilt of 150 days old. None of the twelve farrow to wean farms and none of the 23 pools (in eighteen different farms) collected from breeding animals older than one year (sows and boars) resulted NoV positive. A second sampling stage, carried out between October 2018 and January 2019; focused on NoV positive areas identified during the first stage and in fattening animals. Thirty-seven swine herds were sampled (27 fattening and ten farrow to finish) and 100 faecal samples collected of which 70 from fattening and 30 from farrow to finish farms. The swine herds were located in five out of seven Veneto provinces: thirteen in Padua, eight in Treviso, eight in Vicenza, four in Verona and four in Venice (Fig. 1). Such sampling approach identified fifteen out of 100 faeces samples as NoV positive (15%), collected in eleven out of 37 swine herds, of which 9/27 fattening and 2/10 farrow to finish farms. All but one swine herd were positive to the GII.P11 P-type. The GII.P18 was detected in a fattening farm located in Padua province in animals of four months of age (Fig. 1), exclusively in 2018. The observation of any seasonality in the distribution of NoVs was beyond the scope of the study, therefore not assessed.

**Table 1:** Univariable analysis of NoV detection characteristics.

Characteristic	Category	Positive farms, % (No. pos/total)	p-value
Farms	Fattening	37.2 (16/43)	0.06
	Farrow to wean	0.0 (0/10)	
	Farrow to finish	28.6 (6/21)	
GII.P11 NoV genotype	Fattening	75.0 (12/16)	0.119
	Farrow to finish	100.0 (6/6)	
NoV detection (No. pools)	Animals aged <90 days	3.0 (1/33)	0.015
	Animals aged >90 days	17.9 (28/156)

### NoVs detection in swine is associated with the age

No significant association between NoV detection and herd typology was identified (Fisher’ test: p-value = 0.06), although the p-value tends towards a statistical significance. No farrow to wean farms was positive, while the NoV detection proportion was higher for fattening farms (37.2%) and slightly lower for farrow to finish farms (28.6%). In addition, NoV detection for fattening and farrow to finish farms was significantly associated with age (one-sided z-test: p-value = 0.015). The NoV detection proportion for animals aged >90 days old (17.9%) was significantly higher compared to animals aged <90 days old (3.0%). No significant association between GII.P11 NoV P-type and herd typology was found (p-value = 0.119) (Table 1 and Supplementary Table S1).

### The Italian swine GII NoVs display a high RdRp genetic diversity

NoV positive samples (n = 27) were subjected to Sanger sequencing to obtain a larger RdRp gene fragment compared to sequences available and the full VP1 sequence, as two samples presented low levels of positivity. An 800 bp fragment, corresponding to the terminal part of the RdRp (4200–5080 (5′-3′)) was obtained for twelve out of 27 NoV positive samples, by contrast, no sequences were obtained for the VP1, using previously published primer pairs^10^. A discordant result was observed between the shorter (300 bp) and the larger (800 bp) RdRp sequences of two samples, hence excluded from the phylogenetic analysis. BLAST analysis of the 300 bp sequences showed the highest similarity with GII.P11 viruses, by contrast the 800 bp sequences showed the highest similarity with GII.P18 viruses. Twenty-eight Italian swine NoV RdRp sequences underwent phylogenetic analyses, of which 25 generated in the present study and three obtained in previous studies, two of 1,687 bp and 286 bp^29^ and one of 258 bp^12^. The phylogenetic analysis was conducted including 124 RdRp sequences of which 97 retrieved from Genbank. In details, two sequences retrieved from Genbank were of about 1,550 bp, fourteen of around 800 bp in length, and 81 sequences between 150 and 350 bp.

**Figure 2:**
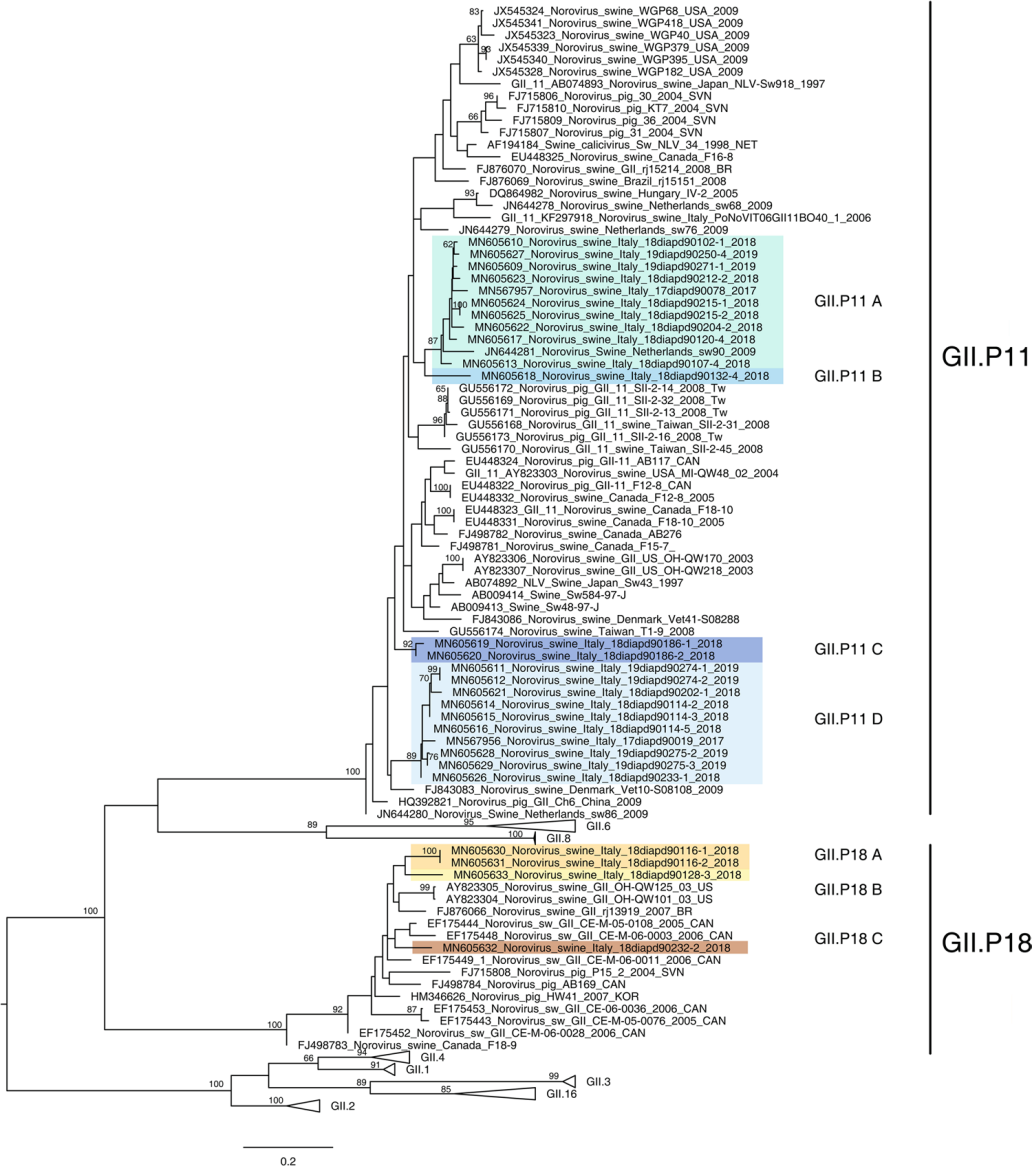
Phylogenetic analysis based on the RdRp nucleotide sequence. Maximum likelihood phylogenetic tree was obtained using PhyML 3.0. The subgroups of the Italian viruses described in this paper were highlighted in different colours. Numbers at the nodes indicate the bootstrap support values. Bootstrap values lower than 60% were omitted.

The phylogenetic analysis of the RdRp gene showed that NoVs identified in pigs fall into two distinct P-types: GII.P11 and GII.P18, revealing a co-circulation of two P-types. In detail, 23 out of 27 NoV samples belong to the GII.P11 P-type and the remaining 4 to the GII.P18 P-type. The Italian swine GII.P11 and GII.P18 strains show a high degree of genetic variability (Fig. 2). Viruses, belonging to both P-types, appear distinctly grouped and within each P-type distinct genetic subgroups were identified. Such grouping was supported by p-distance values: samples belonging to the same subgroup share nucleotide (nt) identity greater than 90% (Table 2). Following this criterion, the GII.P11 Italian NoVs appear to form four distinct genetic subgroups, namely: A, B, C, D (Fig. 2). The inter subgroup similarity among the four Italian GII.P11 genetic subgroups ranged between 81.07% and 89.35%, while the intra subgroup similarity ranged between 92.12% and 100% (Table 2). In detail: the GII.P11A subgroup is composed by ten NoVs identified between 2017 and 2019 detected in six provinces (Padua, Rovigo, Treviso, Vicenza, Verona and Venice); the GII.P11B by one NoV identified in 2018 in Verona province; the GII.P11C by two NoVs identified in 2018 in the same farm located in Treviso province and the GII.P11D by ten NoVs identified in seven different farms between 2017 and 2019 located in four different provinces (Padua, Treviso, Venice and Vicenza) (Fig. 2).

**Table 2:** Percent avarage similarity values and standard deviation of GII.P11 and GII.P18 swine Italian NoV subgroups, at nucleotide and amino acidic levels.

GII.P11	A		B		C		D	
	nt	aa	nt	aa	nt	aa	nt	aa
Group A, mean ± sd (Number of observations)	96.14 ± 1.39 (45)	99.3 ± 0.87 (45)	—	—	—	—	—	—
Group B, mean ± sd (Number of observations)	86.68 ± 0.8 (10)	99.04 ± 0.32 (10)	NA	NA	—	—	—	—
Group C, mean ± sd (Number of observations)	85.76 ± 1.4 (20)	96.84 ± 0.96 (20)	87.37 ± 0.04 (2)	97.88 ± 0.01 (2)	98.38 ± 0 (1)	99.01 ± 0 (1)	—	—
Group D, mean ± sd (Number of observations)	86.32 ± 1.22 (100)	98.33 ± 0.75 (100)	82.09 ± 0.79 (10)	99.89 ± 0.32 (10)	85.86 ± 1.08 (20)	98.08 ± 0.54 (20)	97.37 ± 1.52 (45)	99.54 ± 0.72 (45)
**GII.P18**	**A**		**B**		**C**			
	**nt**	**aa**	**nt**	**aa**	**nt**	**aa**		
Group A, mean ± sd (Number of observations)	NA	NA	—	—	—	—		
Group B, mean ± sd (Number of observations)	87.5 ± 0 (2)	100 ± 0 (2)	NA	NA	—	—		
Group C, mean ± sd (Number of observations)	86.2 ± 0 (2)	100 ± 0 (2)	87.8 ± 0 (1)	98.53 ± 0 (1)	NA	NA		

Note: Each observation is the percent similarity calculated for a pair of sequences. NA indicates that no tests were calculated due to low number of sequences to compare. —Indicates that the comparison of the two subgroups is reported in another cel.

Italian GII.P18 swine NoVs cluster in three distinct subgroups (Fig. 2). The GII.P18 subgroups are composed by 2018 sequences; viruses belonging to each individual subgroup were identified in one single Veneto province (Padua, Treviso, Vicenza) (Fig. 2). The GII.P18A subgroup is composed by two sequences (MN605630_Norovirus/swine/Italy/18diapd90116-1/2018 and MN605631_Norovirus/swine/Italy/18diapd90116-2/2018) of the same farm located in Treviso province; the GII.P18B by one NoV identified in Vicenza province and the GII.P18C by one NoV identified in Padua province (Fig. 2). The p-distance values among the Italian swine GII.P18 subgroups range between 86.20% and 87.80% (Table 2). The other available GII.P18 sequences from swine are from U.S.A., Brazil, Canada and Slovenia identified between 2004 and 2007.

The GII.P11 and GII.P18 P-types co-circulated in swine in three Veneto provinces: Padua, Treviso and Vicenza.

### The swine Italian GII NoVs present unique RdRp amino acid mutations

**Figure 3:**
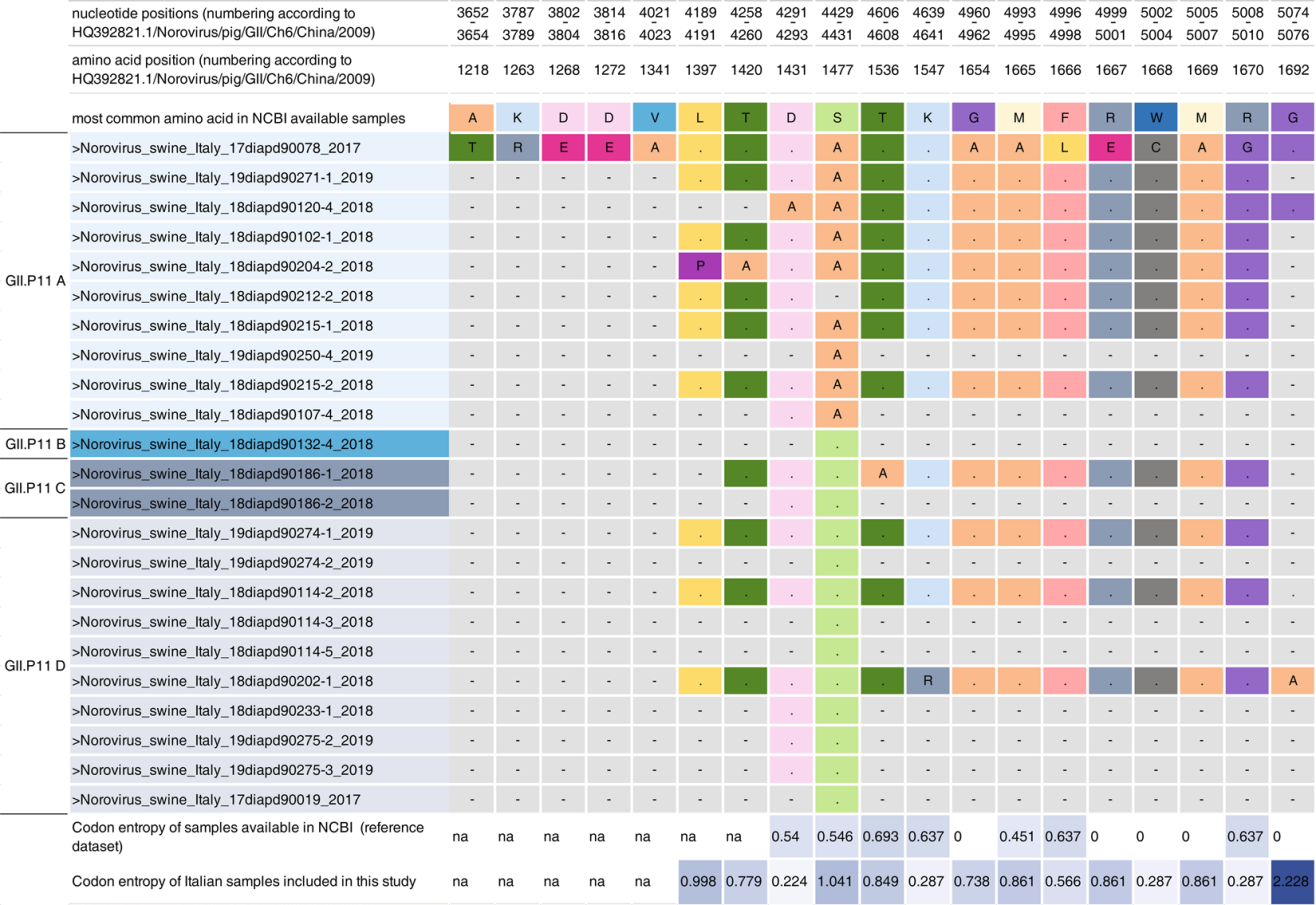
Unique amino acid mutations of Italian GII. P11 RdRp sequences. The figure displays the unique aa mutations of the Italian GII.P11 RdRp sequences, not present in any other GII sequences. The reference sequence used for the aa and nt numbering was: HQ392821.1_Norovirus/pig/GII/Ch6/China/2009. Mutations are reported with the one letter amino acid code. Dots are used to indicate the presence of the same aa for each sequence maintaining the same colour, while dashes indicate that genetic information in that position is not available. The last two rows reported the codon entropy calculated for the Genbank available GII.P11 NoVs and for the Italian GII.P11 samples described in this study. Colour intensity reflects entropy values. Na = not attributable.

To further analyse the molecular characteristics of the Italian swine GII NoVs, we compared the deduced amino acid (aa) sequences with those available in Genbank. Eighteen unique aa mutations, or rather not present in any other GII.P11 viruses available in Genbank, were identified and distributed in Italian swine GII.P11 NoVs belonging to GII.P11A (n = 10), C (n = 1) and D (n = 3) subgroups, while no unique aa mutations were observed for the GII.P11B viruses (Fig. 3). Five unique aa mutations proved to be common between the three subgroups, while thirteen specific aa mutations were identified in each subgroup (GII.P11A n = 10, GII.P11C n = 1 and GII.P11D n = 2). Within the GII.P11A subgroup, the virus identified in 2017, Norovirus/swine/Italy/17diapd90078/2017 (MN567957), presented two aa mutations not common to the other GII.P11A subgroup viruses: F1666L and R1667E (Fig. 3). Interestingly, twelve unique aa mutations were observed for one GII.P11A identified in 2017 (MN567957_Norovirus/swine/Italy/17diapd90078/2017 when compared only to the Norovirus/pig/GII/Ch6/China/2009 GII.P11 sequence with which shared the higher identity (HQ392821.1) (Fig. 3), excluding the aa mutation present in all GII.P11A viruses (S1477A) and in one non Italian NoV (JN 644281_1_ Norovirus_ Swine_Netherlands_sw90_2009). The remaining six unique aa mutations were possessed by four GII.P11 NoVs belonging to the GII.P11A (n = 3), GII.P11C (n = 1) and GII.P11D (n = 2) subgroups.

The aa mutation S1477A was identified in  nine out of ten GII.P11A viruses representing a polymorphism of this subgroup; it was shared by only one NoV European strain of swine origin (JN644281.1_Norovirus/Swine/Netherlands/sw90/2009) (Fig. 3). Some Italian swine GII.P11 strains presented single aa mutations different to each other, with two NoVs detected in 2018 presenting two aa mutations (Fig. 3). The L1397P and T1420A mutations were possessed by one GII.P11A (MN605622_Norovirus/swine/Italy/18diapd90204-2/2018) and the K1547R and G1692A by one GIIP.11D (MN605621_Norovirus/swine/Italy/18diapd90202-1/2018).

By comparing the deduced aa sequences of the swine GII.P18 available in the public databases, the Italian swine GII.P18 NoVs presented three unique aa mutations, here described for the first time: R1429K, present in the MN605633_Norovirus/swine/Italy/18diapd90128-3/2018 strain, the I1395V and T1420A in the MN605632_Norovirus/swine/Italy/18diapd90232-2/2018 strain. In addition, in order to further analyse the aa variability observed in the swine Italian GII.P11 NoVs, the aa positions of the unique aa mutations were analysed for their entropy levels. Briefly, the entropy analysis was conducted comparing the swine GII.P11 NoVs identified in the present study and GII.P11 sequences available in Genbank, which were used as a reference dataset as well. The following viruses were included: 23 GII.P11 nt sequences collected in Italy between 2017 and 2019 (21 generated in this study,  two from our previous study^29^ and 44 GII.P11 NoVs, detected worldwide (Fig. 3).

Eight out of eighteen aa positions where unique mutations were identified in the Italian GII.P11 viruses, displayed higher entropy values. Nonetheless, 4/18 aa positions, presenting unique aa mutations (D1431A, K1547R, F1666L, R1670G) showed lower entropy values compared to the reference dataset (Fig. 3).

## Discussion

Our study provided a set of new information on GII NoVs circulating in the animal reservoir, representing the only study available on swine NoV conducted over one year, in one of the major swine production areas in Italy. The most recent studies available on swine NoVs in Europe dates back to 2011^30^. The NoV prevalence detected in the present study was equal to 11.4%, in agreement with previous studies conducted in Europe (<0.5–18.9%)^8,18,19^. The different prevalence values for swine NoV in Europe could be explained by differences in sampling strategies, ages of pigs sampled, housing and management practices, detection methods and/or primers specificities. Indeed, beyond the universal primer pairs (p290-p110) used for NoV detection in samples of animal origin, several studies have proposed different primer pairs to increase the sensitivity for NoV detection^14,17,18,23^.

Our study, stratified per age groups, showed that fattening animals (>90 days of age) were the animal category identified as positive in the majority of cases. This data is in agreement with previous studies, which showed that swine NoVs were detected mainly, if not exclusively, in fattening animals^8,13,16,20,30^. The reason why such animals present a higher probability to be NoV positive needs further investigation. However, several studies reported the presence of NoV in other age groups such as suckling pigs^13,19,31^, suggesting that additional surveys should be planned to confirm the NoV prevalence in sows, gilts, boars and fattening animals (< and >90 days old). All NoV positive samples were detected in healthy pigs. Such evidence confirms previous studies that identified NoV in pigs without clinical signs^9,16,17^, although in China, NoV was reported in pigs presenting diarrhoea^15,31,32^. Nevertheless, the aim of the present study was not to assess the correlation between NoV detection and clinical signs.

Interestingly, the different sampling strategy, on farm, adopted for the present study, compared to our previous survey at the slaughterhouse conducted in the same geographical area^29^, allowed the detection of a higher number of NoV positive swine farms. Of note, the two sampling approaches adopted by our group, at slaughterhouses^29^ and on farm (this study) surveyed a similar number of swine farms: 74 and 79, respectively, with the on-farm strategy identifying a higher number of positive farms: 22/79 compared to 2/74 at slaughterhouses. This study allowed the identification of two genotypes commonly detected in swine GII.P11 and GII.18^14,15,18,19,21^.

However, this study represents the first report of GII.P18 in swine in Italy, and the third in Europe since it was previously identified in Slovenia and Germany^19,32^. The GII.P18 was observed solely in 2018; nonetheless, our previous study, conducted at slaughterhouses in 2017^29^ suggested the circulation of the GII.P18 in North East Italy evidencing a possible recombination event between GII.P11 and GII.P18. The co-circulation of two P-types paired with the genetic variability observed in the Veneto region poses questions regarding the introduction and evolution of NoVs in North East Italy. Two samples tested positive for both GII.P11 and GII.P18, depending on the method used for p-typing^10,29,33^, and were consequently ruled out from the phylogenetic analysis. This inconsistency of outcomes might be related to the affinity of the methods employed toward a specific p-type, a phenomenon previously observed^17^. However, these findings are suggestive of the occurrence of NoV coinfections in the Italian swine population and of great interest, since recombination events between different genotypes might lead to the emergence of viruses with different genetic and antigenic properties in comparison to their parental strains^34,35^.

The present study discloses newly and recently identified GII.P NoV sequences from the animal reservoir of longer length compared to those available from swine using a primer-walking approach developed previously^29^. In fact, 100 RdRp sequences from swine are available in GenBank of which only eleven longer than 310 bp, highlighting the paucity in genetic information available on swine NoVs and the disparate dataset available to conduct phylogenetic analysis due to the variety of the RdRp sequence lengths.

The swine GII.P11 and GII.P18 Italian NoVs, which are further divided into distinct genetic subgroups with an inter-subgroup maximum nucleotide (nt) similarity not higher than 90%, are composed by viruses detected from 2017 to 2019. Every year, the co-circulation of distinct genetic groups within each P-type identified was observed, although further sampling with a higher geographic extension may capture additional and different genetic characteristics than those described so far in Italy. Such observation may lead to the hypothesis that the Italian swine NoV genetic subgroups may represent distinct and new introductions into Italy every year; however, the circulation of an undetected common ancestor for the Italian swine NoVs cannot be ruled out. The absence of genetic data collected in Italy and Europe between the first study available (2009) and our studies (2017–2019) makes it puzzling to draw any robust conclusions on the origin of the Italian NoVs and the relationship that might exist among them. To our knowledge, this is the first study able to report such high genetic diversity among swine NoVs in a geographically limited area such as the Veneto region in North East Italy.

The genetic diversity observed for the Italian swine GII NoVs was further supported by the several unique aa mutations identified in the RdRp sequences for both P types identified, observed in a higher number for the GII.P11 strains than the GII.P18 ones, and here described for the first time. The genetic variability, linked to these aa positions in the Italian GII.P11 subgroups, was further investigated through the Shannon entropy evidencing that in the aa positions where the mutations occurred, higher entropy values were identified in comparison to the corresponding values of the reference dataset so far available in public database, suggestive of higher probability of codon sequence modification. The Shannon entropy is a valuable tool used to estimate the aa mutation rate of influenza strains^36^, but never used for Noroviruses. Our entropy analysis further supports the uniqueness of the aa mutations identified in the present study.

The Norovirus/swine/Italy/17diapd90078/2017 strain presented the higher number of aa mutations among the GII.P11 viruses but if compared with GII.P11A viruses it presented only two unique aa mutations explaining the high phylogenetic similarity with GII.P11A viruses.

Interestingly, the aa substitution S1477A, shared by almost all GII.P11A strains, occurred within the highly conserved motif B of the RdRp. This motif folded in a loop structure and it is known to assist the correct positioning of the template nucleotide within the active site of the protein and to facilitate the pairing of the incoming rNTP with the templating base^37^. Mutations within motif B proved to increase human NoVs replication rate *in vitro* without affecting the mutation rate^38^. However, the impact of this specific mutation on viral replication in swine remains unclear. The remainder of the unique aa changes were detected in the palm and thumb regions between catalytic motifs; but the impact of mutations occurring in between the catalytic motifs on polymerase fidelity and recombination potential has yet to be determined. The impact of RdRp mutations remains a matter to be further investigated, in particular attention should be devoted on the modified viral fitness they might have^39^.

From a virological point of view, the analysis of a larger fragment of the RdRp gene in comparison to available studies^12,13,18,19^ revealed a high genetic variability of swine NoVs in North East Italy, therefore suggesting that efforts should be made in the generation of longer RdRp swine NoV sequences which are more informative. Clearly, next generation sequencing (NGS) methods, allowing full genome or nearly full genome sequencing, represents the ideal approach, as proven for human NoVs^40–42^ and animal NoVs^43^. Whether these methods could be applicable for swine NoV, should be tested in future studies. However, the typing method proposed here seems to represent an appropriate tool to increase the discriminatory power to resolve the genetic diversity of NoVs with good accuracy, to investigate NoVs populations and the emergence of new variants.

The inability to obtain any VP1 gene sequence for NoVs positive samples identified in the present study might be attributed to low quality and integrity of extracted RNA, which negatively affect the outcome of any molecular‐based technique, especially if the size of the amplicon is very large as in this case^29^. In fact, RNA extraction from faeces is challenging due to the high content of contaminants as reported in numerous studies^44–46^. To a lesser degree, it might be also related to a low degree of identity between the primers used and their target sequences on Italian swine NoVs.

Our data suggest that the densely populated swine areas might become hotspots for NoVs recombination and that intensified monitoring in those areas may shed light on swine NoV epidemiology.

The molecular epidemiology of caliciviruses in animals is largely unexplored, therefore limited information is available regarding the presence and the distribution of swine NoVs in Europe and globally. The role of animals in the ecology of NoVs remains to be established and of interest to understand the interplay between humans and animals for such infection. The available studies on swine NoVs^12,13,15,17–20,23,30^ are single and isolated scientific efforts, lacking continuity and making data fragmentary. It is therefore uttermost necessary to implement harmonized surveillance and research activities, either in swine, or in other animal species, to obtain information on NoV prevalence and the implications of its circulation in the animal reservoirs for possible generation of recombinant variants with zoonotic potential. Due to their global spread, understanding the mechanisms of evolution of GII/GII.P NoVs in the animal reservoir will surely provide valuable information on the evolution pathways of NoVs. Retrospective studies may also contribute in covering the existing gap of knowledge on animal NoVs trough screening of available collections of swine faeces sampled in the past for other scientific purposes.

If NoVs have the potential for zoonotic transmission, the detection of circulating strains and tracking their biological characteristics, will be important in limiting the potential for future epidemics. Moreover, investigating whether and how shedding levels of swine NoVs relate to the occurrence of gastroenteritis in the exposed personnel will aid in implementing risk-mitigating actions for zoonotic transmission. The present study represents a real effort to put the One Health concept in practice, investigating the presence and distribution of NoVs which represent the leading pathogen of gastroenteritis in humans and for which little information is available on the animal host species spectrum and its ecology. Scientific research in the veterinary virology sector is often limited to pathogens that cause epizootic and/or notifiable disease. In a One Health perspective, the study of animal viruses that infects humans, although non-pathogenic for animals, should receive more attention and therefore funding.

## Material and Methods

### Study design, sampling method and study locations

The study area comprised one region of North East Italy: Veneto. The sampling activities were performed between January 2018 and January 2019.

Seventy-four farms were enrolled in this study. Farms were conveniently selected mainly in areas with a high density of pig farms (primarily located in Verona, Padua and Treviso provinces) and with at least 150 heads upwards, in order to collect and test the presence of NoV in pigs of different ages. One faeces pool was collected from two to four swine pens in each swine farm, corresponding to a total of ten to sixteen animals.

A two-stage sampling strategy was applied based on on-farm sampling practice. The first stage (January-June 2018) aimed at determining the NoV detection rate in Veneto swine herds, taking into account all productive categories and age groups present (sows, gilts and fattening pigs from twenty days to 9–10 months of age, before slaughtering); in total 125 swine faeces pools were sampled.

The second stage (October 2018-January 2019), was tailored to consolidate the data obtained during the first sampling stage, as only fattening animals (>90 days of age), housed in fattening and farrow to finish swine herds located in those areas resulted positives during the first stage, were sampled, for a total of 100 swine faeces pools.

In order to represent graphically the estimated density distribution of the swine population, a Kernel Density map was generated using ArcMap 10.5.1. The density map was obtained considering the distribution of swine farms over the Veneto Region territory with a radius of 500 m. The algorithm of Kernel Density Tool develops a smoothly curved surface that is fitted over each farm. The value is highest at the location of the farm and decreases increasing the distance from the farm, reaching zero at the radius distance from the farm. The volume under the surface equals the maximum number of pig places available in the farm. The map was styled using Natural Breaks classification with five classes to have an immediate representation of the density on the territory: dark color means high presence of farms with high values of number of pig places available.

### Statistical analysis

To assess the association between NoV detection and the herd typology, the Fisher’s exact test was used, being one of the expected frequencies <5. Post-hoc pairwise comparisons were then performed using adjusted alfa. Fisher’s exact test was also used to assess the association between NoV P-type and positive herd typology and between NoV detection and animal age. For the first and second analysis, herd was the statistical unit. In the other case, pool faeces were considered as the statistical unit. Statistical significance was set to p < 0.05. All statistical analyses were performed using STATA v.12 (Stata Corp. LP, Texas, USA).

### Molecular detection of swine Norovirus

#### Sample preparation

Each faecal sample was diluted 1:5 (w/v) in Phosphate Buffered Saline (PBS) supplemented with antibiotics (10,000 IU/ml of penicillin G, 10 mg/ml of streptomycin, 5,000 IU/ml nystatin, 0.25 mg/ml gentamicin sulphate). Faeces homogenates were diluted in a final 20% glycerol solution (v/v) (Sigma Aldrich, St Louis, MO, USA) as preserving agent, vortexed and centrifuged at +4 °C for 5 minutes (min) at 14,000× *g*. The supernatant was then aliquoted in 2 ml tubes and stored at −80 °C until use.

#### RNA extraction and RT-PCR

One hundred and forty microliters (µl) of faeces homogenate supernatant was used for viral RNA extraction using the “QIAamp Viral RNA mini kit” (QIAGEN, Hilden, Germany) according to the manufacturer’s instructions. The eluted RNA was used for a one-step RT-PCR by using primer pair p290 and p110 and “Super Script III One-Step RT-PCR System kit with Platinum Taq DNA polymerase” kit (Invitrogen, Carlsbad, CA, USA). The p290-110 primer pair targets a 300 bp fragment of the RdRp gene conserved in the *Caliciviridae* family^10,33^.

The one-step RT-PCR was carried out in 25 µl reaction volume consisting of water (5 µl), Buffer (12.5 µl), primer (1 µM), Rnase inhibitor (20U), enzyme mix (1 µl), and the extracted RNA (5 µl). The PCR program used, had the following thermal profile: retro-transcription at 55 °C for 30 min; initial denaturation at 94 °C for 2 min, followed by 35 cycles with denaturation at 94 °C for 30 seconds (sec), annealing at 48 °C for 30 sec, extension at 68 °C for 20 sec and final elongation at 68 °C for 5 min. The PCR products were analysed by acrylamide gel (7%) after silver staining. Amplicons of the expected size (300 bp) were Sanger sequenced using the same primers pair to differentiate NoV from other *Caliciviridae* members. Sequences obtained were subjected to BLAST analysis.

### RdRp and VP1 sanger sequencing

Amplification and Sanger sequencing of a larger fragment of the RdRp and VP1 gene were carried out on NoV positive samples as previously described^10,29^. Briefly, one-step RT-PCR was carried out using “SuperScript III One-Step RT-PCR System kit” with Platinum Taq DNA polymerase (Invitrogen, Carlsbad, CA, USA) following thermal profile was applied: retro-transcription at 50 °C for 60 min, initial denaturation at 94 °C for 2 min followed by 40 cycles with Taq activation at 94 °C for 15 sec, annealing at 55 °C for 30 sec, extension at 68 °C for 90 sec and final elongation at 68 °C for 5 min.

The sequences obtained were compared with those available using BLAST (https://blast.ncbi.nlm.nih.gov/Blast.cgi), and nt sequences were deposited into GenBank under accession numbers: MN605609-MN605633.

#### Phylogenetic analysis

Nucleotide sequences of the RdRp gene were compared with those available in Genbank. Sequences were aligned with the MAFFT online software version 7 (https://mafft.cbrc.jp/alignment/software/) and the alignment manually corrected in MEGA 6.0. The best substitution model was identified using the model selection function implemented in MEGA 6.0. The phylogenetic analysis was then performed with PhyML 3.0, setting the parameters of the substitution model indicated by MEGA; to estimate the robustness of the obtained topology, a bootstrap analysis with 100 replicates was performed. The phylogenetic tree was then edited using FigTree v 1.4.3 and coloured with Adobe Illustrator. The p-distances of the nt RdRp sequences were calculated using MEGA 6.0.

#### Identification of unique amino acid mutations and sequence entropy analysis

Presence of unique aa mutations of the RdRp sequences, generated in the present study, was investigated.

The sequence entropy was calculated to evaluate the genetic variability between the GII.11 P NoVs identified in swine faecal samples collected in Italy between 2017 and 2019, and the GII.P11 sequences available in Genbank, considered as a reference dataset.

The entropy was calculated only for specific RdRp aa positions, corresponding to the unique aa positions identified in the Italian dataset. The entropy in each aa position was calculated as the sum of the entropy values of the three nucleotide positions that make the corresponding codon and provides measure of the probability of a codon sequence modification. The Shannon entropy of each nucleotide position was calculated using the HIV Los Alamos National Laboratory Entropy-Two tool (https://www.hiv.lanl.gov/content/)^47,48^.

## Supplementary information

Supplementary information.

## Data Availability

All data generated or analysed during this study are included in this published article (and its Supplementary Information Files).
