# Author Correction: Unrevealed genetic diversity of GII Norovirus in the swine population of North East Italy

**DOI:** 10.1038/s41598-020-69599-3

**Published:** 2020-07-22

**Authors:** L. Cavicchio, L. Tassoni, A. Laconi, G. Cunial, L. Gagliazzo, A. Milani, M. Campalto, G. Di Martino, M. Forzan, I. Monne, M. S. Beato

**Affiliations:** 1 Diagnostic Virology Laboratory, Department of Animal Health, Istituto Zooprofilattico Sperimentale delle Venezie (IZSVe), Viale dell’Università 10, 35020 Legnaro, Padua Italy; 2 EU, OIE/FAO and National Reference Laboratory for Avian Influenza and Newcastle Disease, Istituto Zooprofilattico Sperimentale delle Venezie (IZSVe), Viale dell’Università 10, 35020 Legnaro, Padua Italy; 3 Epidemiology Department, Istituto Zooprofilattico Sperimentale Delle Venezie (IZSVe), Viale dell’Università 10, 35020 Legnaro, Padua Italy; 4 Department of Veterinary Virology, University of Pisa, Viale delle Piagge 2, 56124 Pisa, Italy; 5 Department of Comparative Biomedicine and Food Science, University of Padua, Legnaro, Padua Italy

Correction to: *Scientific Reports* https://doi.org/10.1038/s41598-020-66140-4, published online 08 June 2020

This Article contains a formatting error in Figure 1 which makes the key unreadable. The correct Figure 1 appears below.

**Figure 1:**
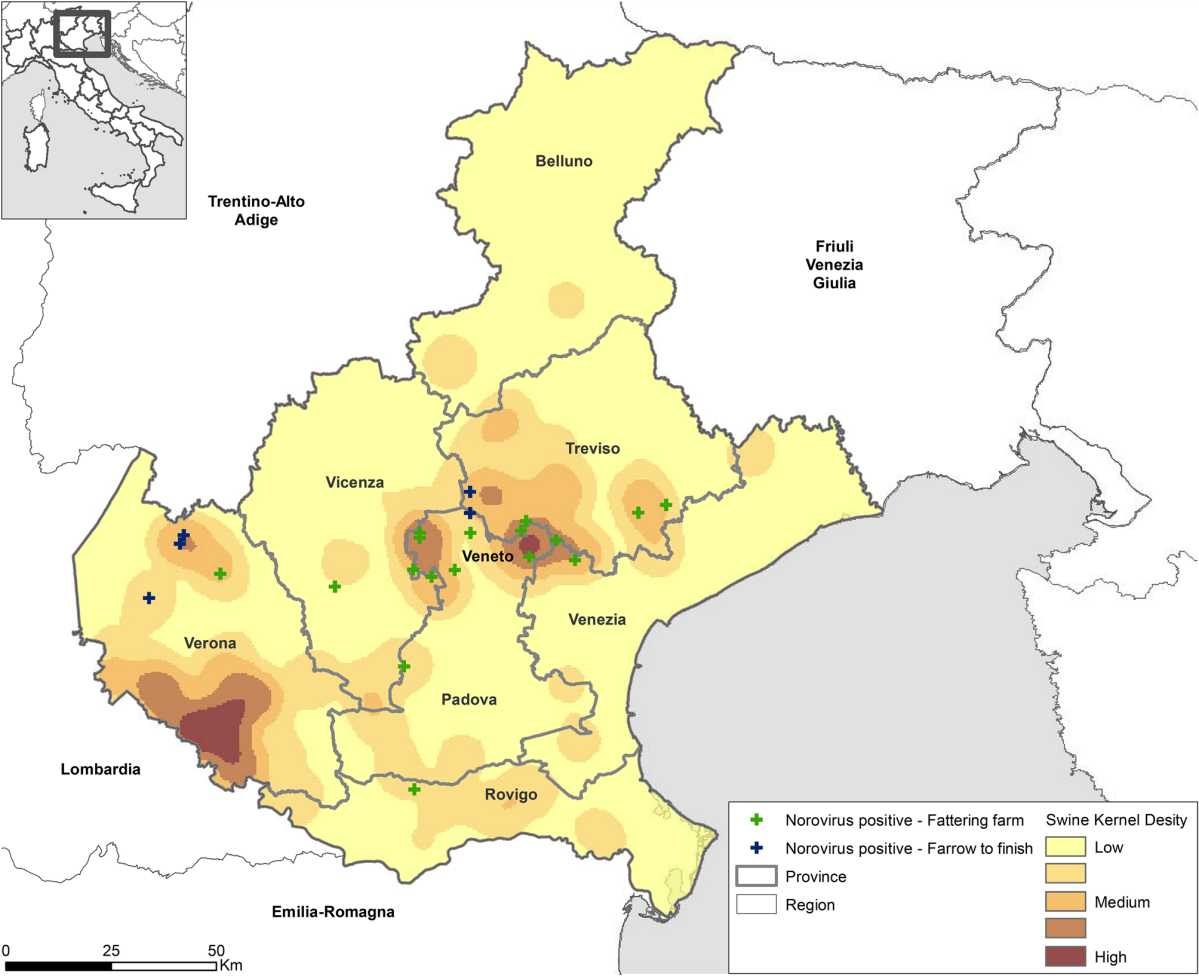
Density map (Kernel Density) of Veneto swine farms and distribution of NoV positive swine farms: 2018–2019. Green crosses identify the NoV positive fattening farms, dark blue crosses the NoV positive farrow to finish farms. The Kernel Density tool calculates the density of features in a neighbourhood around those features. The input data was the industrial swine farms in Veneto region and the density was calculated considering the potential capacity of each farm. The parameter used to calculate the density was a radius of 500 m and the raster cell of the analysis was 10 km^2^.

