# Supplementary material for: Unrevealed genetic diversity of GII Norovirus in the swine population of North East Italy
Source: Sci Rep. 2020 Jun 8;10:9217. doi: 10.1038/s41598-020-66140-4 (PMC7280493; doi:10.1038/s41598-020-66140-4)
Supplement: Supplementary file 1 — Supplementary information. [file 41598_2020_66140_MOESM1_ESM.docx]

**Unrevealed genetic diversity of GII Norovirus in the swine population of North East Italy**

L. Cavicchio^1§^, L. Tassoni^1§^, A. Laconi^2a^, G. Cunial^3^, L. Gagliazzo^3^, A. Milani^2^, M. Campalto^1^, G. Di Martino^3^, M. Forzan^4^, I. Monne^2^, M.S. Beato^1*^

^1^ Diagnostic Virology Laboratory, Department of Animal Health, Istituto Zooprofilattico Sperimentale delle Venezie (IZSVe), Viale dell’Università 10, 35020, Legnaro, Padua, Italy

^2^ EU, OIE/FAO and National Reference Laboratory for Avian Influenza and Newcastle Disease, Istituto Zooprofilattico Sperimentale delle Venezie (IZSVe), Viale dell’Università 10, 35020, Legnaro, Padua, Italy

^3^ Epidemiology Department, Istituto Zooprofilattico Sperimentale Delle Venezie (IZSVe), Viale dell’Università 10, 35020, Legnaro, Padua, Italy

^4^ Department of Veterinary Virology, University of Pisa, Viale delle Piagge 2, 56124, Pisa, Italy.

^a^ Department of Comparative Biomedicine and Food Science, University of Padua, Legnaro, Padua, Italy

^§^These authors have equally contributed

* Corresponding author: Maria Serena Beato, email: msbeato@izsvenezie.it

**Supplementary Table S1. Univariable analysis of characteristics on detection of NoV.**

|  | | | **Farms** | | | **NoV detection, % herds (n.)** | | | **GII.P11 NoV genotype, % herds (n.)** | | **Faecal pools** | | | **NoV detection,  % pools (n.)** | |
| --- | --- | --- | --- | --- | --- | --- | --- | --- | --- | --- | --- | --- | --- | --- | --- |
| **Stage, month and year of sampling** | | | **Fattening** | **Farrow to wean** | **Farrow to finish** | **Fattening** | **Farrow to wean** | **Farrow to finish** | **Fattening** | **Farrow to finish** | **Animals aged < 90 days** | **Animals aged > 90 days** | **Other** | **Animals aged < 90 days** | **Animals aged > 90 days** |
| **1st stage** | Jan-Jun | 2018 | 32 | 10 | 20 | 40.6 (13) | 0 (0) | 25.0 (5) | 69.2 (9) | 100.0 (5) | 33 | 121 | 36 | 3.0 (1) | 18.2 (22) |
| **2nd stage** | Oct-Jan |  |  |  |  |  |  |  |  |  |  |  |  |  |  |
|  |  | 2019 | 11 | 0 | 1 | 27.3 (3) | 0 (0) | 100.0 (1) | 100.0 (3) | 100.0 (1) | 0 | 35 | 0 | 0 (0) | 17.1 (6) |
| **Total** | | | 43 | 10 | 21 | 37.2 (16) | 0 (0) | 28.6 (6) | 75.0 (12) | 100.0 (6) | 33 | 156 | 36 | 3.0 (1) | 17.9 (28) |
| ***Overall*** | | | 74 | | | 29.7 (22) | | | 81.8 (18) | | 255 | | | 11.4 (29) | |
| ***p-value*** | | | - | | | 0.06 | | | 0.119 | | - | | | 0.015 | |
